# Diagnostic Performance of AIMS65, Glasgow-Blatchford, and Pre-endoscopy Rockall Scores in Predicting Clinical Outcomes Among Upper Gastrointestinal Bleeding Patients in the Emergency Department

**DOI:** 10.7759/cureus.96795

**Published:** 2025-11-13

**Authors:** Nuurul Huda Nik Pa, Afliza Abu Bakar, Dazlin M Sabardin, Azlan Helmy Abd Samat

**Affiliations:** 1 Emergency Medicine, Hospital Canselor Tuanku Muhriz, Universiti Kebangsaan Malaysia, Kuala Lumpur, MYS

**Keywords:** aims65 score, emergency department services, gastrointestinal endoscopy, glasgow-blatchford score, mortality prediction, risk assessment, rockall score, transfusion requirement, upper gastrointestinal hemorrhage

## Abstract

Introduction: Acute upper gastrointestinal bleeding (UGIB) requires immediate risk stratification in emergency departments (EDs) to optimize resource utilization and patient outcomes. Several validated scoring systems are available for this purpose. The Glasgow-Blatchford Score (GBS) integrates clinical and laboratory parameters to predict the need for urgent intervention or blood transfusion. The pre-endoscopy Rockall score relies solely on clinical variables such as age, hemodynamic status, and comorbidities to estimate risk before endoscopy. The AIMS65 combines five easily obtainable, clinical, and laboratory variables: albumin, international normalized ratio, altered mental status, systolic blood pressure, and age, providing a simple and objective tool for early mortality prediction, which can be rapidly applied in the ED. However, the comparative effectiveness of these tools in predicting diverse outcomes among Malaysian ED patients remains unclear. This study compared the performance of AIMS65, GBS, and pre-endoscopy Rockall scoring systems in predicting clinical interventions and outcomes among patients with acute UGIB presenting to the ED in Malaysia.

Methods: A retrospective cohort study of 293 adult UGIB patients who presented to the ED of a single tertiary academic hospital from January to December 2022 was conducted. Patients were identified using International Classification of Diseases, 10th Revision (ICD-10) coding and were scored using all three systems. Primary outcomes were early blood transfusion (≤24 hours), endoscopy (≤24 hours), ICU admission, rebleeding, and in-hospital mortality. Discriminative performance was assessed using receiver operating characteristic curves with area under the curve (AUC) analysis, and optimal cut-off values were determined.

Results: Among 293 patients (median age: 70 years, 60.4% male), GBS demonstrated good performance for predicting early transfusion (AUC: 0.830, 95% CI: 0.782-0.871) and fair performance for ICU admission (AUC: 0.666, 95% CI: 0.609-0.720). AIMS65 showed fair performance for mortality prediction (AUC: 0.717, 95% CI: 0.661-0.768). Pre-endoscopy Rockall demonstrated variable performance (AUC: 0.510-0.667). All systems performed poorly in predicting early endoscopy and rebleeding (AUC: <0.60).

Conclusion: For transfusion prediction, GBS achieved good performance (AUC: 0.830), while mortality prediction was best achieved using AIMS65 (AUC: 0.717). All scoring systems showed limited utility for predicting endoscopy timing and rebleeding. Score selection should therefore be tailored to specific clinical decisions in emergency UGIB management.

## Introduction

Upper gastrointestinal bleeding (UGIB) poses major clinical challenges in emergency departments (EDs) worldwide, with an annual incidence of 15-172 per 100,000 adults [1,2] and mortality rates of 8%-14% despite therapeutic advances [3,4]. Emergency physicians must rapidly assess patients ranging from hemodynamically stable individuals to those in hemorrhagic shock, making immediate risk stratification critical for optimizing resource allocation, guiding blood transfusion decisions, intensive care unit (ICU) admission, and endoscopic intervention timing [5,6]. In resource-constrained settings such as the ED in Malaysia, the absence of standardized risk stratification protocols represents a crucial gap in emergency care, where clinical decisions often rely on physician judgment rather than validated prediction tools. 

The Glasgow-Blatchford Score (GBS), AIMS65, and pre-endoscopy Rockall (pre-Rockall) scores are among the most widely studied risk stratification tools for the management of UGIB [3,5,7-9]. The GBS incorporates both clinical and laboratory parameters and has demonstrated strong performance in identifying patients who require intervention while safely facilitating outpatient management of low-risk cases [3,10]. The AIMS65 score offers distinct advantages for ED application, using five readily available clinical and laboratory variables: albumin <3.0 g/dL, international normalized ratio (INR) >1.5, altered mental status, systolic blood pressure (SBP) ≤90 mmHg, and age ≥65 years that are typically included in the initial ED workup. It can be rapidly calculated without the need for endoscopic data, allowing early prognostication and identification of patients at higher risk of in-hospital mortality and adverse outcomes [8,9]. In contrast, the pre-Rockall score enables immediate risk assessment at presentation using only clinical variables: age, hemodynamic status, and comorbidities before endoscopic evaluation [9,11].

Despite the potential clinical utility of these scoring systems, their comparative performance in predicting the need for early intervention and clinical outcomes has not been adequately evaluated in the context of Malaysian healthcare. Differences in patient demographics, healthcare infrastructure, and clinical practices may influence the predictive accuracy of these tools. Comparative studies in the local settings would provide invaluable guidance for the selection of the most appropriate scoring criteria.

Our primary objective was to evaluate the discriminative performance of AIMS65, GBS, and pre-Rockall scores across multiple clinically relevant endpoints among patients with acute UGIB presenting to the ED in Malaysia, which included (1) need for early interventions (blood transfusion within 24 hours, endoscopy, and ICU admission), and (2) clinical outcomes (rebleeding, early mortality, and in-hospital mortality). Secondary objectives included evaluating optimal cut-off values for each endpoint. 

The 24-hour threshold for early endoscopy was selected in accordance with international consensus recommendations that advocate endoscopic evaluation within 24 hours of presentation for all UGIB patients [10]. Moreover, a large, randomized trial found no mortality benefit from endoscopy performed earlier than 6 hours, supporting the clinical relevance of the 24-hour definition [12]. This approach also aligns with our institution’s protocol for acute UGIB management.

## Materials and methods

Study design and setting

This retrospective cohort study was conducted at an ED of a tertiary academic hospital in the capital city, Kuala Lumpur, Malaysia. The hospital is a mixed adult and pediatric urban academic teaching hospital with approximately 70,000 annual patient visits. The study included patients who visited the ED from January 1, 2022 to December 31, 2022.

Study population

All adult patients (≥18 years) with clinical evidence of acute UGIB admitted through the ED between January and December 2022 were screened for inclusion. Potential UGIB cases were initially identified from hospital admission records using International Classification of Diseases, 10th Revision (ICD-10) diagnostic codes (Table 1). Each case was then cross-verified to determine the source of admission; only patients admitted directly from the ED were included, and to confirm data accuracy, duplicate records were excluded.

**Table 1:** ICD-10 codes for UGIB case identification ICD-10 diagnostic codes are used to identify potential UGIB cases from hospital administrative data. These codes were applied to screen 536 probable UGIB cases, of which 362 were confirmed as true UGIB after clinical review, and 293 met final study inclusion criteria (Figure 1). Codes encompass variceal bleeding, peptic ulcers, hemorrhagic gastritis, Mallory-Weiss syndrome, and non-specific GI bleeding presentations. GI, gastrointestinal; ICD-10, International Classification of Diseases, 10th Revision; UGIB, upper gastrointestinal bleeding.

ICD-10 code	Diagnosis
I85.0	Esophageal varices with bleeding
I85.9	Esophageal varices without bleeding
I186.4	Gastric varices
I198.2	Esophageal varices without bleeding in diseases classified elsewhere
I198.3	Esophageal varices with bleeding in diseases classified elsewhere
K22.6	Gastroesophageal laceration-hemorrhage syndrome
K25.0	Gastric ulcer acute: with hemorrhage
K25.1	Gastric ulcer: acute with perforation
K25.2	Gastric ulcer acute: with hemorrhage and perforation
K25.3	Gastric ulcer acute: without hemorrhage and perforation
K25.4	Gastric ulcer: chronic or unspecified with hemorrhage
K25.5	Gastric ulcer: chronic or unspecified with perforation
K25.6	Gastric ulcer: chronic or unspecified with both hemorrhage and perforation
K25.7	Gastric ulcer: chronic without hemorrhage or perforation
K25.9	Gastric ulcer: unspecified as acute or chronic without hemorrhage or perforation
K26.0	Duodenal ulcer: acute with hemorrhage
K26.2	Duodenal ulcer: acute with both hemorrhage and perforation
K26.3	Duodenal ulcer acute: without hemorrhage and perforation
K26.4	Duodenal ulcer: chronic or unspecified with hemorrhage
K26.5	Duodenal ulcer: chronic or unspecified with perforation
K26.6	Duodenal ulcer: chronic or unspecified with both hemorrhage and perforation
K26.7	Duodenal ulcer: chronic without hemorrhage or perforation
K26.9	Duodenal ulcer: unspecified as acute or chronic without hemorrhage or perforation
K27.0	Peptic ulcer, site unspecified: acute with hemorrhage
K27.1	Peptic ulcer, site unspecified: acute with perforation
K27.2	Peptic ulcer, site unspecified: acute with both hemorrhage and perforation
K27.3	Peptic ulcer, site unspecified: without hemorrhage and perforation
K27.4	Peptic ulcer, site unspecified: chronic or unspecified with hemorrhage
K27.5	Peptic ulcer, site unspecified: chronic or unspecified with perforation
K27.6	Peptic ulcer, site unspecified: chronic or unspecified with both hemorrhage and perforation
K27.7	Peptic ulcer, site unspecified: chronic without hemorrhage or perforation
K27.9	Peptic ulcer, site unspecified: unspecified as acute or chronic without hemorrhage or perforation
K28.0	Gastrojejunal ulcer: acute with hemorrhage
K28.1	Gastrojejunal ulcer: acute with perforation
K28.2	Gastrojejunal ulcer: acute with both hemorrhage and perforation
K28.3	Gastrojejunal ulcer: without hemorrhage and perforation
K28.4	Gastrojejunal ulcer: chronic or unspecified with hemorrhage
K28.5	Gastrojejunal ulcer: chronic or unspecified with perforation
K28.6	Gastrojejunal ulcer: chronic or unspecified with both hemorrhage and perforation
K28.7	Gastrojejunal ulcer: chronic without hemorrhage or perforation
K28.9	Gastrojejunal ulcer: unspecified as acute or chronic without hemorrhage or perforation
K29.0	Acute hemorrhagic gastritis
K92.0	Hematemesis
K92.1	Melaena
K92.2	Gastrointestinal hemorrhage, unspecified
K92.8	Other specified diseases of digestive system
K92.9	Disease of digestive system, unspecified

**Figure 1:**
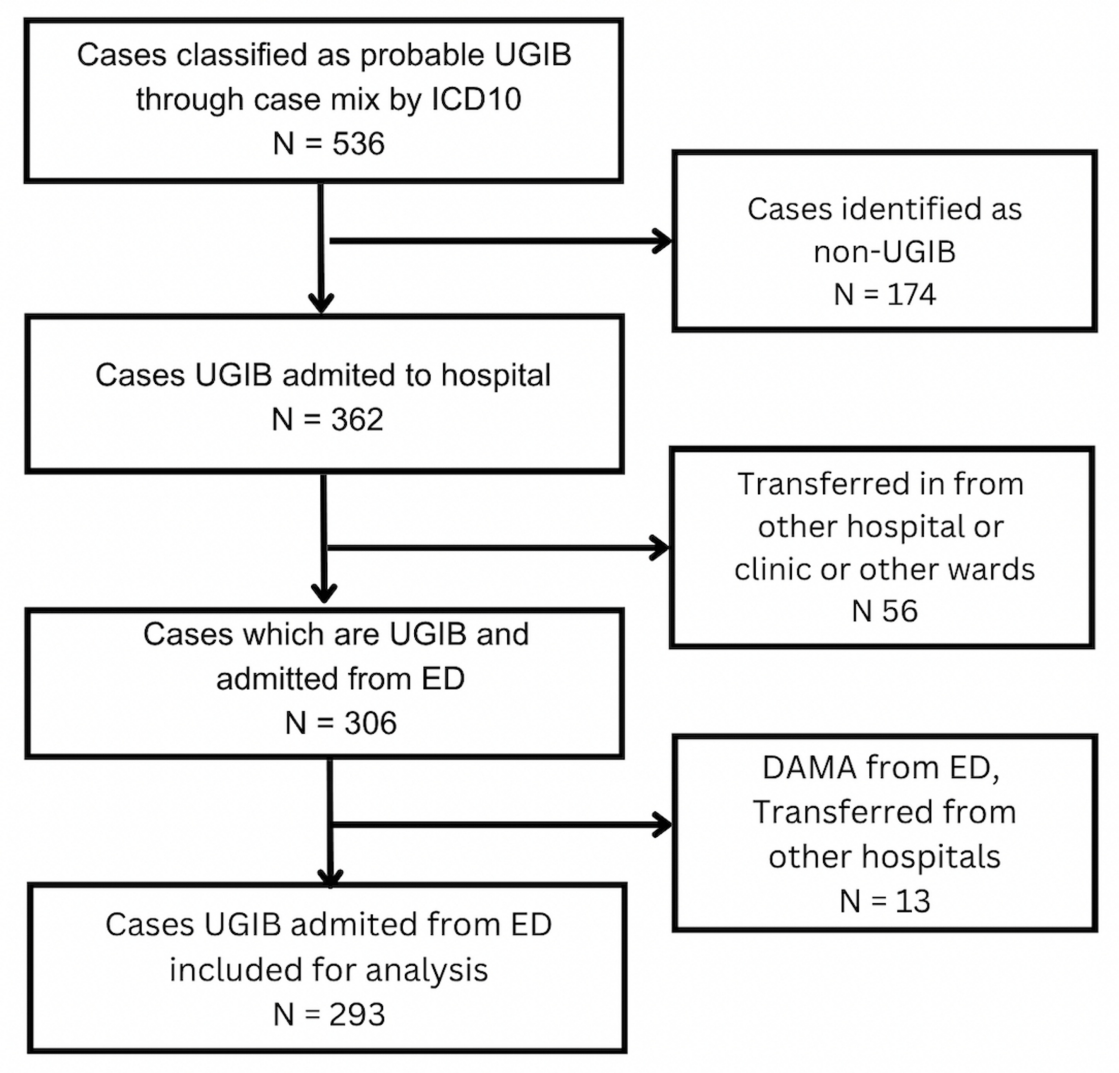
Patient selection flowchart Flowchart showing patient selection process. Of 536 probable UGIB cases identified by ICD-10 diagnostic codes, 174 non-UGIB cases were excluded. Of 362 UGIB cases admitted to the hospital, 56 transferred or readmitted cases were excluded. Of 306 UGIB cases admitted from the ED, 13 were excluded (DAMA or transfers). Final analysis included 293 UGIB patients admitted from the ED. DAMA, discharge against medical advice; ED, emergency department; ICD-10, International Classification of Diseases, 10th Revision; UGIB, upper gastrointestinal bleeding.

All confirmed ED admissions were subsequently reviewed against predefined inclusion and exclusion criteria. Inclusion required presentation with hematemesis, coffee-ground vomitus, melena, or hematochezia with a confirmed UGIB source. Exclusion criteria included lower gastrointestinal bleeding, direct ward or outpatient admissions, inter-hospital transfers, and cases discharged against medical advice. The final dataset comprised 293 eligible ED-admitted UGIB patients (Figure 1).

Hematochezia was included among the initial screening criteria to ensure comprehensive identification of potential UGIB cases, as some patients with brisk upper gastrointestinal hemorrhage may present with fresh per-rectal bleeding. However, during case verification, all patients were reviewed to confirm the bleeding source, and those determined to have a lower gastrointestinal origin were excluded in accordance with the predefined exclusion criteria.

Sample size 

Sample size was calculated using DeLong's method for comparing correlated receiver operating characteristic (ROC) curves, assuming area under the curve (AUC) values of 0.75 (AIMS65) and 0.65 (GBS) [7], with an assumed correlation of 0.6, α = 0.05, and 80% power. This yielded an initial requirement of 312 patients for pairwise comparisons. After applying Bonferroni correction (α = 0.0167) to account for multiple comparisons across three scoring systems, the required sample size increased to 380 patients.

A total of 293 patients were enrolled and included in all analyses. This represents 93.9% of the initial sample size requirement (312 patients) and 77.1% of the Bonferroni-corrected target (380 patients). All 293 patients were included in analyses for AIMS65, pre-Rockall, and GBS. 

Post-hoc power analysis using the actual sample of 293 patients indicated that the study achieved approximately 78% power for individual pairwise comparisons (compared with the planned 80% power) and approximately 70% power after Bonferroni correction. While this represents a moderate reduction from the target power, the achieved sample size remains adequate to detect clinically meaningful differences (≥0.10 in AUC) between scoring systems, consistent with thresholds considered significant in diagnostic accuracy studies. This moderate shortfall slightly increases the risk of Type II error but does not substantially compromise the study's ability to identify clinically relevant differences in discriminative performance. All power analyses were conducted using MedCalc version 23.3.4 (MedCalc Software, Belgium) employing DeLong's method for comparing correlated ROC curves.

Study protocol

UGIB cases were identified from the hospital's case mix system using ICD-10 diagnostic codes for UGIB, including esophageal and gastric varices, peptic ulcers (gastric, duodenal, and gastrojejunal) with or without hemorrhage, hemorrhagic gastritis, gastroesophageal laceration-hemorrhage syndrome, and unspecified gastrointestinal bleeding. The complete list of 52 specific ICD-10 codes used for case identification is provided in Table 1 to ensure reproducibility. Eligible patients were matched with ED records, and demographic, clinical, laboratory, intervention, and outcome data were extracted.

UGIB was defined as bleeding within the oesophagus to the duodenum, manifesting as hematemesis, coffee-ground vomitus, melena, or haematochezia [13]. Primary outcomes included early blood transfusion (within 24 hours of ED presentation), early endoscopy (endoscopic intervention performed within 24 hours in accordance with international guideline recommendations), ICU admission, rebleeding (recurrent hematemesis, melena, or nasogastric aspirate of fresh blood during admission after initial bleeding control), early mortality (death within 48 hours of ED presentation), and in-hospital mortality.

Data completeness: All variables required for calculating the three risk scoring systems, AIMS65 (albumin, INR, altered mental status, SBP, and age), GBS (hemoglobin, urea, SBP, heart rate (HR), melena, syncope, hepatic disease, and cardiac failure), and pre-Rockall score (age, shock status, and comorbidities), were explicitly documented and available in 100% of cases. No imputation or assumption was required for any scoring variable.

For descriptive baseline characteristics not used in risk score calculations, the absence of documentation was interpreted as the feature being absent, consistent with established chart review methodology [14,15], where positive clinical findings are typically recorded. For example, the history of previous endoscopy was not explicitly documented in 58 patients (19.8%) and was recorded as absent for these cases. This approach did not affect risk score calculations or their discriminative performance.

Risk score

The AIMS65 score consists of five variables, albumin <3.0 g/dL, INR >1.5, altered mental status, SBP ≤90 mmHg, and age ≥65 years, each contributing one point for a total range of zero to five [8]. It was developed as a simple bedside tool for mortality prediction. The GBS incorporates hemoglobin, urea, SBP, HR, melena, syncope, hepatic disease, and cardiac failure, producing a score from zero to 23 [3]; higher scores indicate increased need for transfusion or endoscopy, while a score of zero identifies low-risk patients who may be safely discharged. The pre-Rockall score, which ranges from zero to seven, uses only clinical parameters: age, hemodynamic status, and comorbidities, allowing rapid risk assessment before endoscopy [9]. All scoring was performed using the validated MDCalc electronic calculator (MedCalc Software Ltd, Ostend, Belgium; https://www.medcalc.org; 2024) by a single trained researcher to ensure consistency.

Statistical calculations

All analyses were conducted using SPSS version 22.0 (IBM Corp., Armonk, NY) and MedCalc version 23.3.4 (MedCalc Software, Belgium). Descriptive statistics were applied for patient characteristics; continuous variables were summarized as mean ± SD or median (interquartile range or IQR), and categorical variables as frequencies and percentages. Between-group comparisons used Chi-square or Fisher’s exact test for categorical data and t-test or Mann-Whitney U for continuous data, depending on distribution.

ROC curves were generated for AIMS65, GBS, and pre-Rockall scores to assess predictive performance for clinical outcomes. AUC values with 95% confidence intervals (CIs) were calculated and compared using DeLong’s method, with Bonferroni correction applied for multiple comparisons. Optimal cut-offs were identified using Youden’s index, and diagnostic metrics (sensitivity, specificity, accuracy, predictive values, and likelihood ratios) were calculated. A two-sided p<0.05 was considered significant, with p<0.01 after correction.

Ethical considerations

This study complied with the Declaration of Helsinki and was approved by the institutional research ethics committee (JEP-2023-451). Patient confidentiality was protected through anonymization, and informed consent was waived due to the retrospective design and use of de-identified data.

## Results

Study population and baseline characteristics

Clinical data and baseline characteristics are summarized in Table 2. A total of 293 patients who presented to the ED with acute UGIB (median age: 70 years (IQR: 61-78), 60.4% male, n=177) were included in the study. Most patients had underlying hypertension (195, 66.6%), diabetes (159, 54.3%), dyslipidemia (101, 34.5%), and less than a quarter had ischemic heart disease (69, 23.5%) or peptic ulcer disease (70, 23.9%).

**Table 2:** Baseline characteristics of patients with UGIB Demographic and clinical characteristics of study participants (n=293). Data are presented as n (%) for categorical variables and median (IQR) for continuous variables. Median age was 70 years (IQR: 61-78), with male predominance (177, 60.4%). Most patients were triaged as the yellow category (191, 65.0%). Common comorbidities included hypertension (195, 66.6%), diabetes mellitus (159, 54.3%), and dyslipidemia (101, 34.5%). Over one-third (106, 36.2%) had a previous endoscopy. The mode of ED presentation was not available (NA) for 146 patients (49.8%). COPD, chronic obstructive pulmonary disease; ED, emergency department; GERD, gastroesophageal reflux disease; IQR, interquartile range; OGDS, oesophagogastroduodenoscopy; UGIB, upper gastrointestinal bleeding.

Characteristics (N=293)	n (%)
Age in median (IQR)	70 (61-78)
Age group	
18-35 years	13 (4.4)
36-59 years	57 (19.5)
60-80 years	163 (55.6)
>80 years	60 (20.5)
Gender	
Male	177 (60.4)
Female	116 (39.6)
Race	
Malay	128 (43.7)
Chinese	137 (46.8)
Indian	20 (6.8)
Others	7 (2.4)
Mode of presentation to ED	
Private car	106 (36.2)
Ambulance	38 (13)
NA	146 (49.8)
Triage	
Red	62 (21.1)
Yellow	191 (65)
Green	38 (13)
Comorbidities	
Ischemic heart disease	69 (23.5)
Congestive heart failure	12 (4.1)
Renal failure/disease	62 (21.2)
Disseminated malignancy	41 (14)
Peptic ulcer disease/gastritis/GERD	70 (23.9)
Esophageal varices	9 (3.1)
Hypertension	195 (66.6)
Dyslipidemia	101 (34.5)
Diabetes mellitus	159 (54.3)
Cerebrovascular disease	48 (16.4)
Gouty arthritis/osteoarthritis	27 (9.2)
COPD/bronchial asthma	12 (4.1)
Hepatic disease	40 (13.7)
Others	75 (25.6)
History of previous OGDS	106 (36.2)

Clinical characteristics of patients with acute UGIB 

At presentation, the mean SBP was 119.7 ± 25.0 mmHg, HR was 95.4 ± 21.3 beats per minute, and respiratory rate was 20.1 ± 4.0 breaths per minute. The mean hemoglobin was 8.39 ± 2.82 g/dL and mean urea was 15.79 ± 11.92 mmol/L (Table 3).

**Table 3:** Clinical characteristics, interventions, and outcomes of patients with UGIB ALP, alkaline phosphatase; ALT, alanine aminotransferase; APTT, activated partial thromboplastin time; AST, aspartate aminotransferase; GBS, Glasgow-Blatchford Score; GCS, Glasgow Coma Scale; HDU, high dependency unit; HR, heart rate; ICU, intensive care unit; INR, international normalized ratio; IQR, interquartile range; OGDS, Oesophagogastroduodenoscopy; PT, prothrombin time; RR, respiratory rate; SBP, systolic blood pressure; SD, standard deviation; SpO₂, oxygen saturation; UGIB, upper gastrointestinal bleeding.

Clinical characteristics	N (%)	Mean ± SD, median (IQR)
			Vital signs		
SBP (mmHg)		119.71 ± 24.95
HR (beats/minute)		95.42 ± 21.34
RR (breath/minute)		20.13 ± 3.94
Temperature (°Celsius)		36.91 ± 0.63
GCS		14.99 ± 0.06
SpO2 (%)		97.32 ± 4.46
Blood investigations		
Full Blood Count		
Hemoglobin (x10^9^/L)		8.39 ± 2.82
Hematocrit (%)		26.67 ±16.17
Platelet (x10^9^/L)		285.99 ± 127.29
Electrolytes and Renal Profile		
Urea (mmol/L)		15.79 ± 11.92
Creatinine (μmol/L)		187.86 ± 217.45
Sodium (mmol/L)		136.24 ± 5.11
Potassium (mmol/L)		4.44 ± 0.92
Calcium (mmol/L)		2.39 ± 0.16
Magnesium (mmol/L)		0.81± 0.14
Liver Function Tests		
Albumin (g/L)		29.19 ± 6.89
ALT (U/L)		30.16 ± 34.89
AST (U/L)		43.89 ± 71.61
ALP (U/L)		119.29 ± 205.28
Bilirubin (μmol/L)		16.52 ± 27.64
Total protein (g/L)		62.41 ± 10.92
Coagulation Profile		
PT (seconds)		17.46 ± 13.77
APTT (seconds)		35.72 ±12.18
INR		1.32 ± 1.04
Intervention		
Transfusion within 24 hours	215 (73.4)	
Total OGDS	265 (90.4)	
OGDS within 24 hours	182 (62.1)	
OGDS after 24 hours	111 (37.9)	
Surgical interventions	19 (6.5)	
Risk stratification score		
AIMS 65 score		1 (IQR 1,2)
GBS score		11 (IQR 8,13)
Pre-Rockall score		4 (IQR 3,5)
Disposition		
Critical care/ICU/HDU	54 (18.4)	
Ward: medical/surgical	239 (81.6)	
Length of hospital stay		6.87 ± 5.79
Outcome		
Rebleeding	23 (7.8)	
Survive to discharge	279 (95.2)	
Overall in-hospital mortality	14 (4.8)	
Early mortality (within 48 hours)	4 (14)	

For the outcome measures, 73.4% patients required early blood transfusion, 62.1% had early endoscopy, 18.4% required ICU admission, 7.8% had rebleeding, and 4.8% had in-hospital mortality. The median scores for AIMS65, GBS, and pre-Rockall were 1 (IQR: 1-2), 11 (IQR: 8-13), and 4 (IQR: 3-5), respectively.

Scoring system associations with clinical outcomes

Overall, there were variable associations between the scoring system and clinical outcomes. AIMS65 demonstrated significant associations with the need for early blood transfusion (p<0.001), early endoscopy (p=0.025), ICU admission (p<0.001), early mortality (p=0.037) and in-hospital mortality (p=0.001) (Table 4). 

GBS also showed significant associations with the need for early blood transfusion (p<0.001), endoscopy (p=0.003), need for ICU admission (p<0.001), in-hospital mortality (p=0.013) but not with early mortality (p=0.490). In contrast, the pre-Rockall score showed significant associations only with the need for early blood transfusion (p=0.032) and in-hospital mortality (p=0.045). None of the scores showed significant associations with episodes of rebleeding (Table 4).

**Table 4:** Comparison between AIM65, GBS, and pre-Rockall scores, with the need for intervention and outcomes among patients with UGIB (N=293) Data are presented as n (%). p-values are calculated using a ᵃPearson chi-square test or a ᵇFischer's exact test. *p<0.05 is statistically significant. Chi-square test statistics: Early Transfusion: AIMS65 (χ²=13.72, p<0.001), GBS (χ²=71.38, p<0.001), and pre-Rockall (χ²=4.57, p=0.032); Early Endoscopy: AIMS65 (χ²=5.04, p=0.025), GBS (χ²=9.00, p=0.003), and pre-Rockall (χ²=2.94, p=0.086); Rebleeding: AIMS65 (χ²=2.20, p=0.138), GBS (χ²=2.16, p=0.142), and pre-Rockall (χ²=2.54, p=0.111); ICU admission: AIMS65 (χ²=13.45, p<0.001), GBS (χ²=16.51, p<0.001), and pre-Rockall (χ²=3.14, p=0.077); In-Hospital Mortality: AIMS65 (χ²=10.18, p=0.001), GBS (χ²=6.11, p=0.013), and pre-Rockall (χ²=4.02, p=0.045); Early Mortality: AIMS65 (χ²=4.37, p=0.037), GBS (χ²=0.48, p=0.490), and pre-Rockall (χ²=0.52, p=0.473) GBS, Glasgow-Blatchford Score; ICU, intensive care unit; UGIB, upper gastrointestinal bleeding.

Interventions/outcome	Score	Cut-off	Yes (N, %)		No (N, %)		Total (N, %)
Need for early blood transfusion	AIMS65	≤1	21 (9.8)		21 (26.9)		42 (14.3)
		>1	194 (90.2)		57 (73.1)		251 (85.7)
	GBS	≤8	34 (15.8)		52 (66.7)		86 (29.4)
		>8	181 (84.2)		26 (33.3)		207 (70.6)
	Pre-Rockall	≤3	21 (9.8)		32 (41.0)		92 (31.4)
			>3	155 (72.1)		46 (59.0)		201 (68.6)
	Need for early endoscopy	AIMS65	≤2	163 (89.6)			89 (80.2)	252 (86.0)
		>2	19 (10.4)			22 (19.8)	41 (14.0)
GBS		≤7	34 (18.7)			38 (34.2)	72 (24.6)
		>7	148 (81.3)			73 (65.8)	221 (75.4)
pre-Rockall		≤2	8 (7.2)			25 (13.7)	33 (11.3)
		>2	103 (92.8)			157 (86.3)	260 (88.7)
Rebleeding		AIMS65	≤1	9 (39.1)	149 (55.2)			158 (53.9)
			>1	14 (60.9)	121 (44.8)			135 (46.1)
		GBS	≤9	5 (21.7)	100 (37.0)			105 (35.8)
		>9	18 (78.3)	170 (63.0)			188 (64.2)
	pre-Rockall	≤4	13 (56.5)	195 (72.2)			208 (71.0)
			>4	10 (43.5)	75 (27.8)			85 (29.0)
Need for ICU admission	AIMS65	≤2	38 (70.4)	214 (89.5)			252 (86.0)
		>2	16 (29.6)	25 (10.5)			41 (14.0)
	GBS	≤12	23 (42.6)	171 (71.5)			194 (66.2)
		>12	31 (57.4)	68 (28.5)			99 (33.8)
	pre-Rockall	≤4	33 (61.1)	175 (73.2)			208 (71.0)
		>4	21 (38.9)	64 (26.8)			85 (29.0)
	In-hospital mortality	AIMS65	≤2	8 (57.1)	244 (87.5)			252 (86.0)
			>2	6 (42.9)	35 (12.5)			41 (14.0)
GBS		≤12	5 (35.7)	189 (67.7)			194 (66.2)
		>12	9 (64.3)	90 (32.3)			99 (33.8)
pre-Rockall		≤3	1 (7.1)	91 (32.6)			92 (31.4)
		>3	13 (92.9)	188 (67.4)			201 (68.6)
Early mortality		AIMS65	≤2	2 (50.0)	250 (86.5)			252 (86.0)
			>2	2 (50.0)	39 (13.5)			41 (14.0)
	GBS	≤12	2 (50.0)	192 (66.4)			194 (66.2)
		>12	2 (50.0)	97 (33.6)			99 (33.8)
	pre-Rockall	≤2	0 (0.0)	33 (11.4)			33 (11.3)
		>2	4 (100.0)	256 (88.6)			260 (88.7)

AIMS65, GBS, and pre-Rockall scoring system performance in predicting clinical outcome

AUC Analysis of AIMS65, GBS, Pre-Rockall Scoring Systems in Predicting Clinical Outcomes

**Figure 2:**
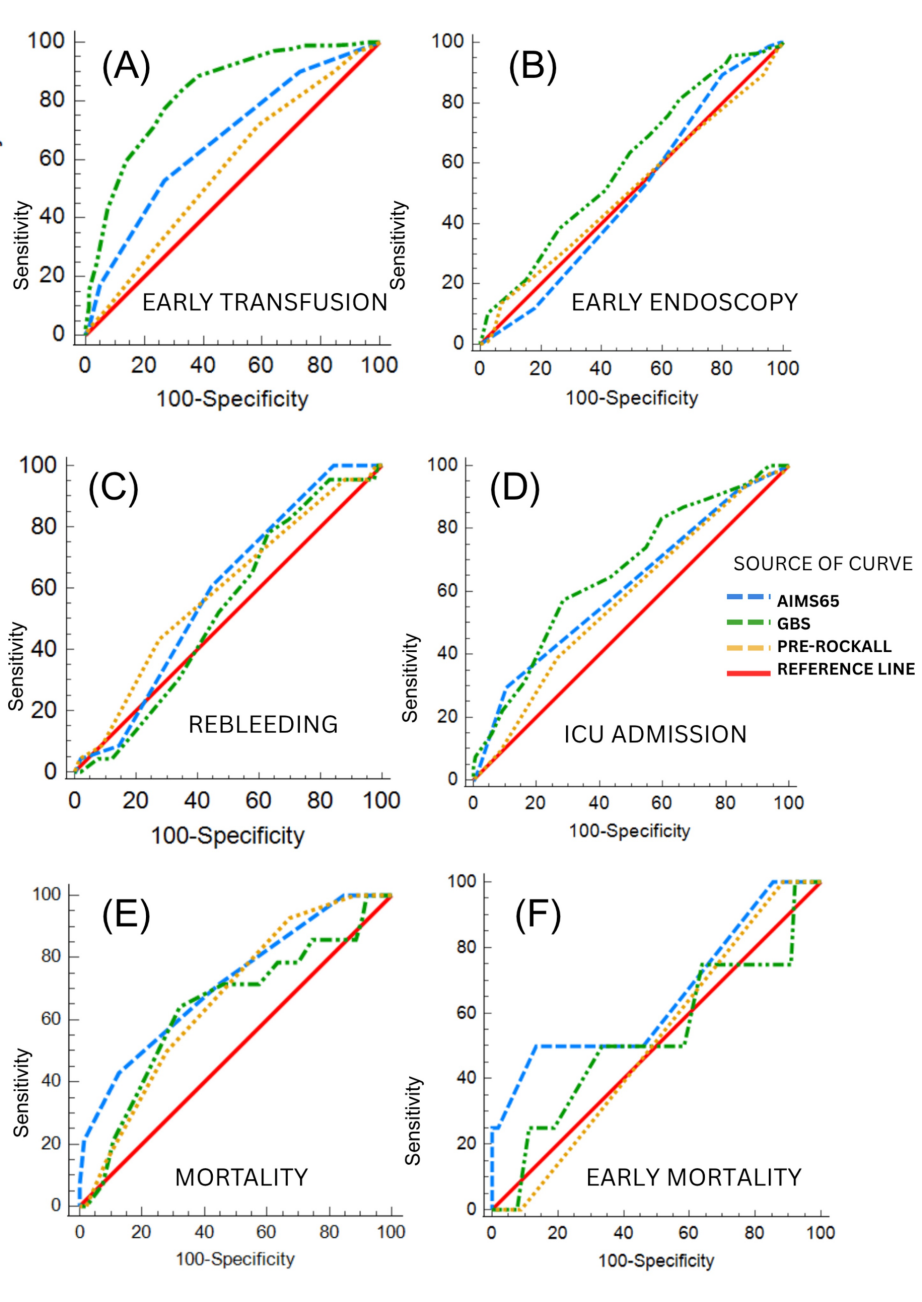
ROC curves for prediction of clinical outcomes: (A) early transfusion, (B) early endoscopy, (C) rebleeding, (D) ICU admission, (E) in-hospital mortality, and (F) early mortality ROC curves comparing AIMS65 (blue dashed line), GBS (green dash-dot line), and pre-Rockall (orange dotted line) scores for six outcomes. The red solid line represents no discrimination (AUC: 0.5). (A) For early transfusion, GBS shows superior performance (AUC: 0.830) than AIMS65 (0.667) and pre-Rockall (0.571). (B) Early endoscopy prediction is poor for all scores (AUC: 0.501-0.602). (C) Rebleeding prediction shows limited utility (AUC: 0.531-0.590). (D) ICU admission shows modest discrimination, with GBS performing best (AUC: 0.666). (E) AIMS65 demonstrates the best performance for in-hospital mortality (AUC: 0.717). (F) Early mortality prediction is limited by low event rate (n=4), with AIMS65 showing an AUC of 0.652. AUC values were calculated using DeLong's method. AUC, area under the curve; GBS, Glasgow-Blatchford Score; ICU, intensive care unit; ROC, receiver operating characteristic.

To predict the need for early transfusion, GBS demonstrated better performance with AUC at 0.830 (95% CI: 0.782-0.871), compared with AIMS65 at 0.667 (95% CI: 0.610-0.721) and pre-Rockall at 0.571 (95% CI: 0.512-0.628). After applying Bonferroni correction for multiple comparisons (α=0.0167), GBS outperformed AIMS65 (AUC difference: 0.163, p<0.0001) and pre-Rockall (AUC difference: 0.259, p<0.0001). Meanwhile, AIMS65 outperformed pre-Rockall (AUC difference: 0.096, p=0.0085) (Figure 2A and Table 5). 

For ICU admission, diagnostic ability was weak to modest, with comparable performance across all the three scores. GBS achieved the highest AUC at 0.666 (95% CI: 0.609-0.720), followed by AIMS65 at 0.616 (95% CI: 0.557-0.672) and pre-Rockall at 0.574 (95% CI: 0.516-0.632), and the differences were not statistically significant (Figure 2D and Table 5).

To predict in-hospital mortality, the AUC of the scores were moderate, ranging from 0.638 to 0.717. AIMS65 demonstrated the best discriminative ability with AUC at 0.717 (95% CI: 0.661-0.768), while pre-Rockall achieved AUC of 0.667 (95% CI: 0.610-0.721) and GBS at 0.638 (95% CI: 0.580-0.693). Pairwise comparisons did not show statistical significance between AIMS65 and the other scores (AUC difference to GBS: 0.41, and AUC difference to pre-Rockall: 0.45) (Figure 2E and Table 5). 

The discriminative ability for AIMS65, GBS, and pre-Rockall scores for predicting the need for early endoscopy, rebleeding, and early mortality (defined as death within 48 hours of ED presentation) was poor with AUC ranging from 0.501 to 0.652 (Figures 2B, 2C, 2F and Table 5).

**Table 5:** AUC analysis and pairwise comparisons for prediction of clinical outcomes AUC values with 95% CI and pairwise statistical comparisons for AIMS65, GBS, and pre-Rockall score across six clinical outcomes in patients with acute UGIB (N=293). AUC quantifies overall discriminative ability, with values interpreted as: 0.5-0.6 (poor), 0.6-0.7 (modest), 0.7-0.8 (moderate), 0.8-0.9 (good), >0.9 (excellent). p-values represent pairwise comparisons of correlated ROC curves using DeLong's method. For early transfusion, GBS (AUC: 0.830) significantly outperformed AIMS65 (0.667, p<0.0001) and pre-Rockall (0.571, p<0.0001). For in-hospital mortality, AIMS65 showed the highest AUC (0.717), though differences were not statistically significant. All scores demonstrated poor discrimination for early endoscopy and rebleeding (AUC: 0.501-0.602). *Statistically significant after Bonferroni correction (p<0.0167). ‡Early mortality results based on low event rate (n=4); interpret with caution. AUC, area under the curve; CI, confidence interval; GBS, Glasgow-Blatchford Score; ICU, intensive care unit; UGIB, upper gastrointestinal bleeding.

Outcome	AUC (95% CI)	p-value of pairwise AUC curves		
		AIMS65	GBS	Pre-Rockall
Early transfusion (n=215)				
AIMS65	0.667 (0.610, 0.721)	-	<0.0001*	0.0085*
GBS	0.830 (0.782, 0.871)	<0.0001*	-	<0.0001*
Pre-Rockall	0.571 (0.512, 0.628)	0.0085*	<0.0001*	-
Early endoscopy (n=182)				
AIMS65	0.501 (0.442, 0.560)	-	0.0769	0.8021
GBS	0.602 (0.543, 0.658)	0.0769	-	0.0932
Pre-Rockall	0.510 (0.451, 0.568)	0.8021	0.0932	-
Rebleeding (n=23)				
AIMS65	0.588 (0.530, 0.645)	-	0.3588	0.9825
GBS	0.531 (0.472, 0.589)	0.3588	-	0.4086
Pre-Rockall	0.590 (0.531, 0.646)	0.9825	0.4086	-
ICU admission (n=54)				
AIMS65	0.616 (0.557, 0.672)	-	0.2951	0.2757
GBS	0.666 (0.609, 0.720)	0.2951	-	0.0506
Pre-Rockall	0.574 (0.516, 0.632)	0.2757	0.0506	-
In-hospital mortality (n=14)				
AIMS65	0.717 (0.661, 0.768)	-	0.4116	0.4502
GBS	0.638 (0.580, 0.693)	0.4116	-	0.7350
Pre-Rockall	0.667 (0.610, 0.721)	0.4502	0.7350	-
Early mortality^‡^ (n=4)				
AIMS65	0.652 (0.694, 0.706)	-	0.5272	0.2472
GBS	0.528 (0.469, 0.586)	0.5272	-	0.8581
Pre-Rockall	0.512 (0.453, 0.571)	0.2472	0.8581	-

Diagnostic Performance Characteristics

Optimal cut-off values and their corresponding diagnostic metrics are summarized in Table 6. Overall, the diagnostic ability of AIMS65, GBS, and pre-Rockall based on the cut-off values varied substantially depending on the outcome measure.

For early transfusion, GBS at a cut-off >8 yielded 84.2% sensitivity (SN) and 87.4% positive predictive value (PPV), and positive likelihood ratio (+LR) of 2.53 with overall diagnostic accuracy of 80%. AIMS65 at a cut-off >1 achieved 53.0% SN, 84.4% PPV, +LR of 1.97, and overall diagnostic accuracy of 73%. For predicting early endoscopy, pre-Rockall demonstrated specificity (SP) of 92.8%, PPV of 75.8%, and +LR of 1.91. However, the overall diagnostic accuracy for all three scores were poor, ranging from 44% to 64%. 

Likewise, the overall diagnostic accuracy for ICU admission was also a modest prediction with GBS (cut-off >12), resulting in 57.4% of SN, 71.6% of SP, negative predictive value (NPV) of 88.1%, and +LR of 2.02. The in-hospital mortality with AIMS65 >2 demonstrated a higher SP of 87.5% compared with GBS and pre-Rockall with SP of 67.7% and 32.6%, respectively, and a +LR of 3.42 (Table 6).

**Table 6:** Diagnostic performance of AIMS65, GBS, and pre-Rockall score at optimal cut-off values Diagnostic performance characteristics of AIMS65, GBS, and pre-Rockall score for predicting clinical outcomes in patients with acute UGIB (n=293). Optimal cut-off values were determined using Youden's index (sensitivity + specificity: -1) to maximize overall discriminative ability. Data are presented as percentages with 95% CI. For early transfusion, GBS >8 showed superior performance (sensitivity: 84.2% and accuracy: 79.5%). For in-hospital mortality, AIMS65 >2 demonstrated the highest specificity (87.5%) and +LR (3.42). All scores showed poor performance for early endoscopy and rebleeding prediction. Early mortality results (n=4) should be interpreted cautiously due to the low event rate. CI, confidence interval; GBS, Glasgow-Blatchford Score; ICU, intensive care unit; NPV, negative predictive value; PPV, positive predictive value; UGIB, upper gastrointestinal bleeding; +LR, positive likelihood ratio; -LR, negative likelihood ratio.

Score	Cut-off value	Sensitivity, % (CI)	Specificity, % (CI)	PPV, % (CI)	NPV, % (CI)	+LR	-LR	Diagnostic accuracy (%)	
Early transfusion									
AIMS65	>1	53.0 (46.1-59.8)	73.1 (61.8-82.5)	84.4 (78.7-88.9)	36.1 (31.7-40.7)	1.97	0.64		73.4
GBS	>8	84.2 (78.6-88.8)	66.7 (55.1-76.9)	87.4 (83.5-90.5)	60.5 (52.0-68.4)	2.53	0.24		79.5
Pre-Rockall	>3	72.1 (65.6-78.0)	41.0 (30.0-52.7)	77.1 (73.3-80.5)	34.8 (27.5-42.9)	1.22	0.68		63.8
Early endoscopy									
AIMS65	≤2	89.6 (84.2-93.6)	19.8 (12.9-28.5)	64.7 (62.2-67.0)	53.7 (39.7-67.1)	1.12	0.53		36.8
GBS	>7	81.3 (74.9-86.7)	34.2 (25.5-43.8)	67.0 (63.5-70.2)	52.8 (42.9-62.5)	1.24	0.55		63.5
Pre-Rockall	≤2	13.7 (9.1-19.6)	92.8 (86.3-96.8)	75.8 (59.4-87.0)	39.6 (37.8-41.5)	1.91	0.93		43.7
Rebleeding									
AIMS65	>1	60.9 (38.5-80.3)	55.2 (49.0-61.2)	10.4 (7.5-14.1)	94.3 (90.8-96.5)	1.36	0.71		55.6
GBS	>9	78.3 (56.3-92.5)	37.0 (31.3-43.1)	9.6 (7.7-11.8)	95.2 (90.1-97.8)	1.24	0.59		40.3
Pre-Rockall	>4	43.5 (23.2-65.5)	72.2 (66.5-77.5)	11.8 (7.5-18.1)	93.7 (91.2-95.6)	1.57	0.78		70.0
ICU admission									
AIMS65	>2	29.6 (18.0-43.6)	89.6 (84.9-93.1)	39.0 (26.9-52.7)	84.9 (82.5-87.1)	2.83	0.79		78.5
GBS	>12	57.4 (43.2-70.8)	71.6 (65.4-77.2)	31.3 (25.1-38.2)	88.1 (84.4-91.1)	2.02	0.60		68.9
Pre-Rockall	>4	38.9 (25.9-53.1)	73.2 (67.1-78.7)	24.7 (18.1-32.7)	84.1 (80.9-86.9)	1.45	0.83		66.9
In-hospital mortality									
AIMS65	>2	42.9 (17.7-71.1)	87.5 (83.0-91.1)	14.6 (8.0-25.3)	96.8 (95.1-98.0)	3.42	0.65		85.3
GBS	>12	64.2 (35.1-87.2)	67.7 (61.9-73.2)	9.1 (6.1-13.3)	97.4 (94.9-98.7)	1.99	0.53		67.6
P re-Rockall	>3	92.9 (66.1-99.8)	32.6 (27.1-38.5)	6.5 (5.5-7.6)	98.9 (93.2-99.8)	1.38	0.22		35.5
Early mortality									
AIMS65	>2	50.0 (6.8-93.2)	86.5 (82.0-90.2)	4.9 (1.8-12.5)	99.2 (97.9-99.7)	3.71	0.58		86.0
GBS	>12	50.0 (6.8-93.2)	66.4 (60.7-71.9)	2.0 (0.8-5.3)	99.0 (97.3-99.6)	1.49	0.75		66.2
Pre-Rockall	>2	100.0 (39.8-100.0)	11.4 (8.0-15.7)	1.5 (1.5-1.6)	98.6 (98.6-98.6)	1.13	0.00		12.6

**Figure 3:**
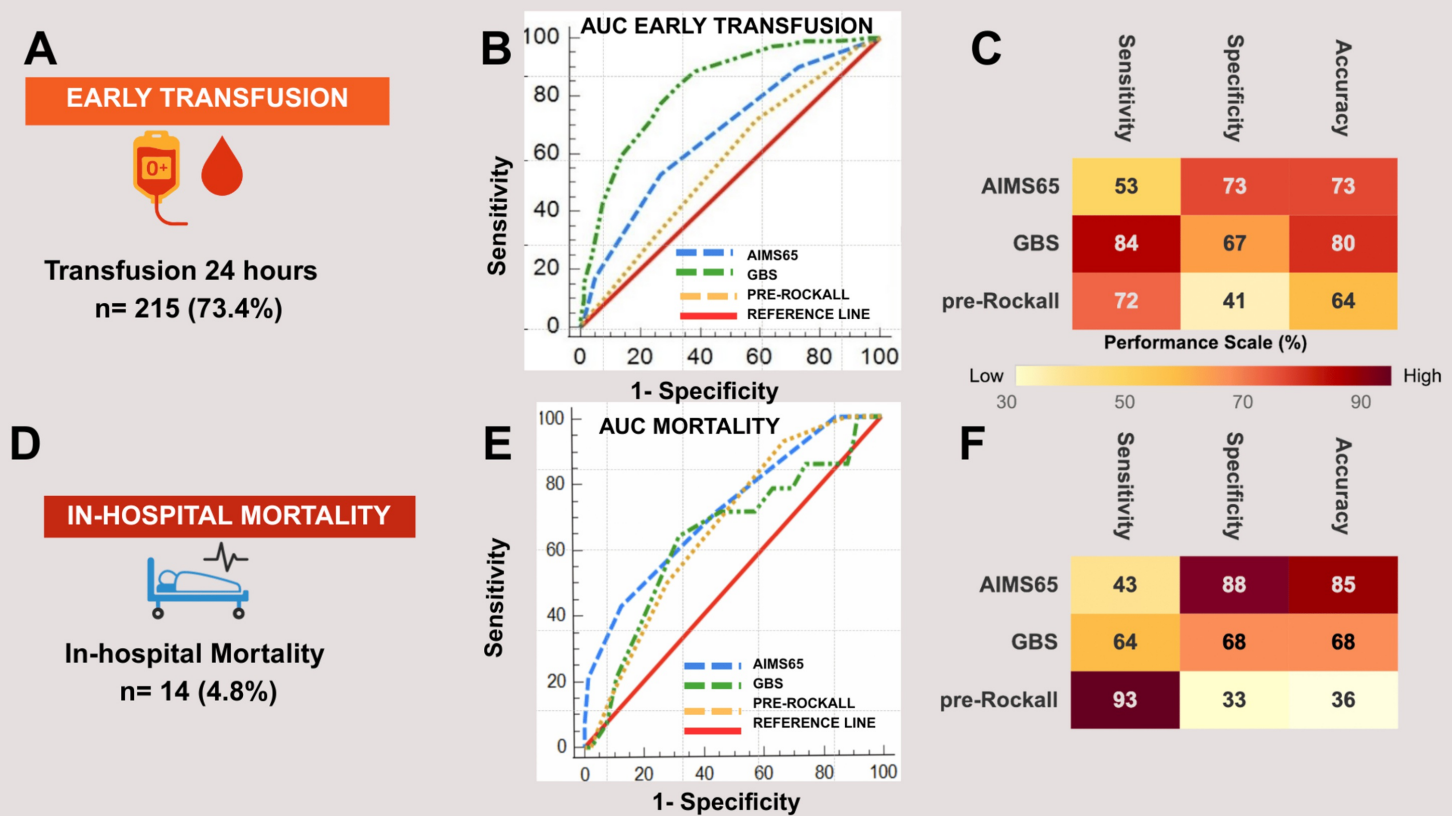
Central illustration summarizing the diagnostic performance of AIMS65, GBS, and pre-Rockall score for early transfusion and in-hospital mortality GBS and AIMS65 demonstrate the best ability to predict the need for early transfusion and in-hospital mortality, respectively. Comparative performance illustration for early transfusion and in-hospital mortality. Panels A and D: outcome prevalence (transfusion: 73.4% and mortality: 4.8%). Panels B and E: ROC curves showing GBS superiority for transfusion prediction (AUC: 0.830, p<0.0001 vs. others) and AIMS65 advantage for mortality (AUC: 0.717, not significant). Red lines = no discrimination. Panels C and F: performance heat maps (yellow-red gradient: 30%-90%) displaying sensitivity, specificity, and accuracy. GBS is optimal for transfusion (sensitivity: 84% and accuracy: 80%); AIMS65 is optimal for mortality (specificity: 85% accuracy). See Table 5 for complete metrics. AUC, area under the curve; GBS, Glasgow-Blatchford Score; ROC, receiver operating characteristic.

Across all evaluated outcomes, GBS exhibited the strongest discriminative performance for early transfusion (AUC: 0.830) and ICU admission (AUC: 0.666). AIMS65 demonstrated moderate diagnostic performance for in-hospital mortality prediction with AUC of 0.717. Conversely, all three scoring tools showed limited discriminative ability for predicting the need for early endoscopy (AUC: 0.501-0.602) and rebleeding (AUC: 0.531-0.590). These findings are highlighted in the central illustration summary in Figure 3.

## Discussion

This study reveals three key findings that may challenge the notion of a universal "best" scoring system for UGIB risk stratification. First, GBS demonstrates good diagnostic performance for predicting early blood transfusion compared with AIMS65 and pre-Rockall score. Second, AIMS65 demonstrates the best diagnostic utility for early and in-hospital mortality among UGIB patients presenting to the ED with an overall diagnostic accuracy of more than 85%. Third, all three systems exhibit variable and limited diagnostic performance across other clinical outcomes, i.e., rebleeding and ICU admission, with no single tool proving superiority over other scores for all clinical outcomes.

GBS discriminates the need for early blood transfusion 

GBS was reliable in discriminating patients requiring early blood transfusion, with a significantly higher AUC of 0.830 compared with AIMS65 (AUC: 0.667) and pre-Rockall (AUC: 0.571). This finding aligns with the original design of GBS, which aimed to identify patients requiring urgent intervention, and is consistent with multiple validation studies showing AUC values ranging from 0.68 to 0.87 for transfusion prediction [3,16]. GBS also demonstrated a reliable ability to detect true positive cases for UGIB patients requiring blood transfusion, evidenced by the relatively high SN, PPV, and moderate +LR. A tool with reliable diagnostic performance may facilitate decision-making among emergency physicians in a busy ED and provide confidence in both identifying transfusion candidates and avoiding unnecessary blood product utilization, a critical consideration in resource-constrained healthcare settings [17].

AIMS65 utility in mortality risk assessment

AIMS65 demonstrated the strongest performance for in-hospital mortality prediction, outperforming both GBS and pre-Rockall, reinforcing its original design purpose for mortality risk stratification. While SN was modest (42.9%), the high SP (87.5%) and +LR of 3.42 may support its utility for prognostic discussions and medical care planning between physicians and families [8,18,19]. This finding aligns with a prospective study of more than 1000 patients by Rout et al., which also identified AIMS65 as the best predictor for mortality [20]. Unlike other scoring criteria, AIMS65 incorporates albumin and INR, which are important markers of systemic illness and liver dysfunction, which may explain the association of AIMS65 with mortality [20]. This is particularly relevant in the context of resource-limited healthcare settings such as Malaysia, where clear communication about mortality risks to family members may influence decision-making and resource allocation [6]. The simplicity of AIMS65, which requires only five routinely available clinical variables, makes it a practical tool for rapid mortality risk assessment in the ED [21].

Variable performance across other clinical outcomes

The diagnostic performance of GBS, AIMS65, and pre-Rockall score was poor and highly variable for other critical outcomes, most notably in detecting the need for endoscopy and rebleeding, evidenced by the poor AUC and overall diagnostic accuracies. This suggests that these scores provide minimal guidance for clinicians in crucial clinical decisions in UGIB management. This pattern reflects a fundamental limitation of static risk stratification tools that rely on presentation variables rather than dynamic clinical response parameters [22]. The poor prediction performance for endoscopy aligns with findings by Farooq et al., who demonstrated that clinical judgment outperformed formal scoring systems for endoscopic intervention timing [23]. Similarly, our rebleeding prediction results suggest that current tools inadequately capture the complex pathophysiology underlying recurrent bleeding, potentially requiring novel biomarkers or dynamic assessment approaches [24,25].

Clinical implementation and optimal cut-off values

The identified optimal cut-offs provide practical guidance for clinical implementation. For transfusion decisions, GBS >8 offers reliable sensitivity (84.2%) with modest specificity (66.7%), aligning with international validation studies showing similar performance [18,26]. For ICU triage, GBS >12 increases specificity to 71.6% but reduces sensitivity to 57.4%, reflecting the need for conservative resource allocation in constrained settings. However, our study showed lower discriminative performance compared with international cohorts, suggesting population-specific validation is necessary [7,16,19,26].

For mortality risk assessment, AIMS65 >2 demonstrates high specificity (87.5%) but modest sensitivity (42.9%), making it suitable for confirming rather than screening high-risk patients. This differs markedly from other validation studies reporting superior sensitivity (85%-88%), indicating AIMS65 may be less effective in populations with higher baseline comorbidity burden [6,16,19,26].

These performance differences likely reflect the unique characteristics of our Malaysian cohort compared with international studies. Our patients were notably older (median age: 70 years vs. 58-65 years in most studies) with substantially higher comorbidity burden; 66.6% had hypertension, 54.3% diabetes, and 21.2% renal disease. These rates were significantly higher than those reported in international cohorts. The relatively poor performance of pre-Rockall (median score: 4 vs. 2-3 in Korean studies) further suggests that clinical variables may have different predictive weights in populations with higher baseline comorbidity and advanced age [7,19,26]. This underscores the importance of population-specific validation and the potential need for locally calibrated models.

From a clinical perspective, these findings suggest that existing scoring systems should complement rather than replace clinical judgment, particularly for time-sensitive decisions such as urgent endoscopy [27]. While GBS and AIMS65 retain value for transfusion and mortality prediction, respectively, they function best as supportive triage tools in resource-constrained emergency settings rather than definitive decision aids. These findings highlight the need for outcome-specific tool selection rather than universal application of a single score. This approach is particularly relevant for Malaysian EDs where resource constraints demand precise risk stratification to optimize blood bank utilization, ICU allocation, and after hours endoscopy services [28,29].

Limitations

This study has several important limitations that warrant consideration. The retrospective, single-center design inherently limits generalizability and introduces potential information bias, as data accuracy was entirely dependent on the completeness and consistency of medical record documentation. While all variables required for risk score calculations were explicitly documented in 100% of cases, certain descriptive baseline characteristics not used in scoring calculations were assumed absent when not documented, following standard chart review methodology. However, this interpretation did not affect risk score calculations, discriminative performance, or any primary study outcomes. Additionally, the restriction to in-hospital mortality without long-term follow-up may have underestimated overall mortality risk, particularly for patients who died shortly after discharge.

The reliance on ICD-10 coding for case identification may have led to the omission of patients with undocumented or miscoded UGIB, potentially underestimating the true case burden. Moreover, as data were collected over a single calendar year (2022) in one center, the findings may not capture year-to-year or seasonal variations in UGIB incidence, case mix, or management practices, thereby limiting external generalizability. Furthermore, the low prevalence of mortality significantly undermines the reliability of NPV and PPV calculations, as these metrics are particularly sensitive to prevalence, thereby limiting the overall predictive performance of the scores. 

Additionally, this study needs to be cautiously interpreted, as it is relatively underpowered, achieving only 78% of the 80% statistical power intended to identify clinically meaningful differences of ≥0.10 in AUC. A prospective, multicenter study would overcome many of these limitations by enabling real-time data collection, standardized assessment protocols, and broader external validity. Such a design could also incorporate additional clinical variables, such as serum lactate, shock index, or organ dysfunction scores, and allow for integration of scoring tools into electronic health records to facilitate automated risk stratification and earlier intervention.

## Conclusions

This study highlights that each UGIB risk score has distinct but limited strengths. The GBS demonstrated good performance for predicting transfusion needs (AUC: 0.830), while AIMS65 showed moderate utility for mortality risk assessment (AUC: 0.717). The pre-Rockall score contributed little additional clinical value. Critically, none of the scoring systems reliably predicted early endoscopy or rebleeding (AUC <0.60). From a clinical perspective, these findings suggest that existing scoring systems should complement rather than replace clinical judgment, particularly for time-sensitive decisions such as urgent endoscopy. While GBS and AIMS65 retain value for transfusion and mortality prediction, respectively, they function best as supportive triage tools in resource-constrained emergency settings rather than definitive decision aids.

The observed performance differences from international validation studies, notably our cohort’s older age (median age: 70 years vs. 58-65 years) and higher comorbidity burden, underscore the importance of population-specific validation and the potential need for locally calibrated models. A context-based approach is therefore recommended: GBS for transfusion triage, AIMS65 for mortality prognostication, and continued reliance on clinical assessment for endoscopy timing and rebleeding risk. Score selection should be tailored to specific clinical decisions rather than applied universally across all aspects of UGIB management.
